# The Role of Basic Psychological Needs in Well-Being During the COVID-19 Outbreak: A Self-Determination Theory Perspective

**DOI:** 10.3389/fpubh.2020.583181

**Published:** 2020-11-12

**Authors:** Dušana Šakan, Dragan Žuljević, Nikola Rokvić

**Affiliations:** Faculty of Legal and Business Studies Dr Lazar Vrtakic, Department of Psychology, Novi Sad, Serbia

**Keywords:** basic psychological needs, well-being, coronavirus disease, Self-Determination Theory, sequential mediation model

## Abstract

Due to the coronavirus outbreak, people around the world are facing various challenges in maintaining their well-being, which can be compromised due to risk of illness and harsh measures of social distancing. As proposed by the Self-Determination Theory, basic psychological needs are essential nutrients of well-being. The aim of this study was to examine the role of basic psychological needs in well-being during the pandemic. A sequential mediation model was examined, that links positive and negative affectivity to well-being (satisfaction with life and general distress) through satisfaction and frustration of the basic psychological needs (for autonomy, competence, and relatedness). The study involved 965 participants (*M*_age_ = 29; 57% females) from Serbia. The Basic Psychological Needs Satisfaction and Frustration scale, Satisfaction with Life Scale, Depression Anxiety Stress Scale 21, and The Serbian Inventory of Affect based on the Panas-X were used. All the tested models were statistically significant. Controlling for age, gender, having children, health, employment, and marital status, direct effects in all models were highly significant, explaining up to 59% of criteria variance. The proportion of the explained variance was even higher when accounting for indirect effects. Sequential mediation models revealed that the indirect relationships between positive and negative affectivity and satisfaction with life and general distress were serially mediated by autonomy satisfaction, competence frustration, relatedness satisfaction, and relatedness frustration. This study raised an important question on how the disposition to experience more positive or negative emotions affects the change in subjective well-being. These results, coherent with the Self-Determination Theory postulates, add to the understanding of human functioning in the times of extraordinary circumstances during a pandemic, by suggesting that satisfaction and frustration of basic psychological needs might have a key role in obtaining optimal well-being.

## Introduction

The new world crisis, caused by the outbreak of the coronavirus, has affected many lives across the globe. Due to its fast transmission, on March 11th, COVID-19 was declared a pandemic by the World Health Organization (WHO). Authorities have quickly responded by enforcing travel bans, quarantines, border closures, curfews, stay-at-home orders, and closures of facilities, including schools, universities, and kindergartens. While all the efforts of the nations are concentrated on resolving epidemiological, clinical, and transmission issues of the COVID-19, mental health issues have largely been misaddressed (1). Studies from previous pandemics, epidemics like SARS, MERS, HIV, etc. identified serious consequences of quarantine such as PTSD along with depressive disorders (2, 3), and several psychiatric comorbidities as depression, anxiety, panic attack, suicidality, and psychotic symptoms (4). Due to serious consequences of the pandemic on mental health, it is crucial to deal with the questions of mental health as soon as possible with first signs of the epidemic outbreak.

Psychological consequences of COVID-19 pandemic are being extensively examined. Early results in China (5, 6) showed increase in negative emotions (such as depression and anxiety) and decrease in satisfaction with life and positive emotions in the general population. Protective factors against anxiety in student population were living in urban areas, cohabitation with parents, and higher family income (5). The risk factor of the experienced anxiety was having contact with an infected person. In the first 2 weeks of the pandemic, greater stress, anxiety, and depression triggered by the pandemic were related with female gender, student status, specific physical symptoms (e.g., myalgia, dizziness, coryza), and poor self-rated health status (7). Since the coronavirus is still an unknown, people with symptoms of coughing, or low fever, as probable signs of flu, can express distress and anxiety, because of the fear of being infected by serious illness (4). Results from Italy proved that youth, women, and people uncertain about the possible COVID-19 infection have reported higher levels of anxiety, distress, sleep disturbances (8), and mood worsening, as well as PTSD symptomatology (9, 10). Similar results were obtained by Favieri et al. (11) also on Italian sample—lower well-being was documented in women, younger than 50 years old, and those with health issues. Also, levels of well-being were lower in people who did not know they were infected, who had direct exposure to the virus, and those who knew affected people (11), as well as in parents who had to balance personal life, work, and raising children (12).

We propose that probable lower well-being during the pandemic arises from the difficulties in satisfying basic psychological needs during quarantine and other proposed measures of fighting against COVID-19.

### Subjective Well-Being

Even though philosophers have been pointing out that happiness is the most powerful promotor of human behavior, it seems that psychologist have been denying its influence for a long time, focusing primarily on negative aspects of human functioning (13, 14). Positive psychology has directed its efforts toward understanding and describing the positive human characteristics that allow the realization of a person's full potential, and along with that, the ability to cope with the everyday challenges (15). One of the biggest contributions to positive psychology is subjective well-being-based research. Well-being is composed of an affective and cognitive-judgmental component (16, 17). The affective component includes the balance between the positive and negative affects (16). Positive affect is related to the degree to which positive emotional states like interest, joy, and trust are felt by a person, while negative affect relates to the degree to which an individual experiences negative emotional states like anxiety, depression, disgust, sadness, and shame (18). The second component of well-being, the cognitive one, satisfaction with life, represents an individual self-evaluation of satisfaction with their life in general (16, 19).

Well-being, often named in the Self-Determination Theory literature as *wellness* (20), is considered thriving or fully functioning, and not only the presence of positive and the absence of negative emotions (20, 21). This concept of well-being is characteristic of the *eudaimonic* approaches of well-being focusing on the fact that full functionality is related to vitality, self-awareness, and self-regulated behavior. The main focus of this approach is on a healthy functioning self, involving the integrated structures, processes as fundamentals of autonomous functioning, rather than those attainments of rewards, status, and esteem (20). Being fully functioning is determined by various factors (22)—such as developmental (e.g., temperament, intellectual capacities, etc.), social (educational opportunities, parental styles), and the political-economic situation (poverty, wars, etc.) (20). Deci and Ryan (23) argued that situational contexts that thwart the satisfaction of the needs diminish well-being. Newer research results have revealed that being fully functional (achieve well-being) depends greatly on some critical events, such as the current global health situation—the COVID-19 pandemic. For example, extreme actions that were introduced to reduce the spread of the virus had influenced the upsurge of poor sleep quality, high distress, and high anxiety (8).

### Positive Affect, Negative Affect, and Their Relations With Satisfaction With Life and General Psychological Distress

Within the science of well-being, positive affect and negative affect are considered two independent constructs, and therefore, they are differently related to other psychological variables. Although not incorporated in any of the personality structure models, these constructs are considered to be traits, or even biobehavioral systems, underlying positive and negative emotional reactions (24–26). For instance, positive affect is positively related to social engagement and favorable events (25), self-efficacy, and resilience (27), while negative affect is related to more frequent negative life events (25), health problems, dysfunctional coping mechanisms (28), and emotional distress (27). Furthermore, extensive research has revealed positive relations between positive affect and satisfaction with life, and the opposite relations between negative affect and satisfaction with life (29–31). Although some studies argued that correlations between satisfaction with life and both negative and positive affects were similar [i.e., (32)], some cross-cultural studies proved the opposite. For example, Kuppens et al. (30) came to a conclusion that when people make judgements about their own satisfaction with life, they take both negative and positive emotions in consideration. However, their results revealed that positive emotions were related to satisfaction with life twice as strongly as the experience of negative affect. As postulated by positive psychology (33), in order to attain greater satisfaction with life, it is necessary to promote the experience of positive emotions, and not solely avoid negative experiences. Also, culture has a moderating effect in relations between affects and satisfaction with life. Positive affect plays a more significant role in accomplishing the “good life” in more developed countries than in countries in which survival values are a priority (30). When basic survival and economic situation are no longer struggling, people can focus more on their self-expression and on the satisfaction of their psychological needs (34) and therefore gain more from their positive experiences.

The presence of unpleasant emotional states is a common consequence of most of the mental health difficulties (35) and one of the most reliable and commonly used indicators of well-being and mental health [e.g., (17)]. One of the dominant theoretical models of the structure and nature of unpleasant emotional states is represented by the Tripartite Model (36). According to this model, general distress is a common feature of both depression and anxiety states (37). On the other hand, there are also factors specific to anxiety and depression which differentiate these states, e.g., somatic tension and increased physiological arousal for anxiety and low positive affect for depression. Various research has demonstrated that depression can be differentiated from other dysfunctional conditions by low positive affect and reduced life satisfaction (27, 38–43). Having in mind that general distress represents a common factor for depression, anxiety, stress, and hyperarousal (44), as well as that the general distress level is quite sensitive to environmental and situational determinants (45), the variation of this construct within the context of the pandemic can be highly anticipated.

### Basic Psychological Needs and Well-Being

As proposed by the prominent and empirically driven Self-Determination Theory (SDT), in order to be fully functional, and to attain well-being, one's basic psychological needs need to be satisfied (need for autonomy, need for competence, and need for relatedness) (20). The need for autonomy is an innate human need for self-endorsed choices, self-volition, and critical thinking (20, 46). The need for competence in a need for having an impact on the environment is a need to perceive self as competent to overcome even difficult obstacles (20, 46). The third need, the need for relatedness, is an intrinsic need for being cared for and to care for others (20, 46).

Recent literature on basic psychological needs has revealed an important distinction between satisfaction and frustrations of needs and designated them as separate concepts (20, 47–55). For instance, one can feel low relatedness due to pandemic-induced quarantine, which diminishes satisfaction with life, but if one feels abandoned by other people, he may feel the thwarting of his relatedness followed by distress and other psychological issues. Thus, frustration of the needs is experienced when social surroundings or events thwart the basic psychological needs of a person. As Vansteenkiste and Ryan (55) pointed out, low need satisfaction does not necessarily imply the frustration of the needs, but, however, need frustration always involves low need satisfaction. In order to attain personal growth satisfaction of the needs is essential, while their frustration is essential to maladaptation and ill-being.

Up until now, it was proven that satisfaction of the basic psychological needs is related to a myriad of positive outcomes. Results of studies have shown that need satisfaction adds to the more autonomous aspects of motivation for learning (20, 46, 54), to vitality (56), positive mood (57), feeling of self-competence (58), lower stress (59), and well-being in general (50, 60–63). This applies vice versa—frustration of the basic psychological needs relates to ill-being and non-functional behaviors (48, 50, 64, 65). Even if it is quite clear that basic psychological needs should be firstly fulfilled in order to attain higher levels of well-being, it still remains a question under which social circumstances. Recent meta-analyses have revealed that major life events (both family and work events such as divorce, retirement, migration, marriage, childbirth, etc.) have effects on affective and cognitive subjective well-being (66). However, so far, it is unknown whether critical events of global proportion like the COVID-19 pandemic have similar effect on well-being as other above-listed life events.

### Basic Psychological Needs During the Coronavirus Pandemic

Satisfaction of the basic psychological needs might be difficult to maintain mostly due to the preventive measures involving social distancing and complete quarantine, which seems to have a very important role in well-being (67). Compelling people to stay at home and not leaving them many choices to make on their own likely caused lower satisfaction of the autonomy and its frustration (68). Furthermore, measures brought left many people without jobs, or working in a non-natural work environment (e.g., remote work from home), and students without their everyday activities in schools and universities, relying on online teaching. All those circumstances could have affected the satisfaction of the need for competence. As for the relatedness satisfaction, it can be satisfied even more due to staying at home with families, but it also can be more thwarted, since staying at home during the pandemic was not a personal choice (autonomously chosen), and thus neither was the way of making close relations with others (indicating relatedness satisfaction).

Studies on the role of basic psychological needs in well-being are limited with regard to acute infectious disease. To our knowledge, no prior work has been done, during previous epidemics (like SARS, HIV, MERS) linking these variables. In the latest literature review during the pandemic of COVID-19, only one study regarding these questions is revealed. Cantarero et al. (69), in examining basic psychological needs effects on well-being, have come to a conclusion that changes in the satisfaction of the three needs mattered to well-being. It turned out that the biggest effect on well-being had the satisfaction of the need for competence, especially when people had the possibility to work as before the pandemic (69). Their further analysis showed that well-being was higher in those participants who made more contact via Internet or phone and was related to the number of days experiencing restrictions. An interesting finding of this study is that autonomy satisfaction was not consistently significant in the prediction of well-being, while needs for relatedness, and, particularly, for competence were constant (69).

### The Present Study

This study is aimed to expand our knowledge of the influence of both satisfaction and frustration of basic psychological needs on well-being in the times of coronavirus which is understood as a thwarting situation or event. The general aim of this research was to examine well-being during the outbreak of COVID-19. Specifically, this study had the aim to examine the relationships between positive and negative affects and satisfaction with life and distress and the role of basic psychological needs as mediators and mechanisms of well-being. Even though there are some studies that treat affects as outcomes of the satisfaction of the basic psychological needs [e.g., (70–72)], our work is more in line with other studies that have treated these variables the other way around and have found that constructs, that are to some extent convergent to affects, serve as predictors for the satisfaction of the basic psychological needs (73–75). We followed the latter setting of variables in the model, because we assumed that people with disposition to feel more positively during the health crisis would be more prone to attain the satisfaction with life, via the satisfaction of the basic psychological needs. Secondly, we expected that people with disposition to feel more negatively, would attain more pronounced distress and less satisfaction with life, via frustration and lower satisfaction of the basic psychological needs. Theoretically, the model of affects used is more a dispositional trait, and basic psychological needs are more behavioral aspects based on dispositional structures, which are sensitive to environmental influences. As discussed above, basic psychological needs might be hard to satisfy due to situational factors incorporated into the government measures for spreading the COVID-19 disease.

Government measures in Serbia regarding the COVID-19 pandemic started by declaring a State of Emergency in the country on March 15th followed by a curfew from 6 p.m. to 5 a.m. introduced on March 19th. At this point, a special government expert body was created to coordinate the national efforts against COVID-19. This body had daily press conferences and informed the nation on the daily number of deaths and newly infected individuals. Addresses by the President of the Republic to the nation followed this press conference almost on a daily basis, and during these addresses, new restrictive measures were announced. Our research began on April 1st, on this day the new measures were announced, the suspension of some business activities such as hairdressers, gyms and fitness centers. Data gathering lasted up until April 10th. At that time, because laboratory capacities were still low, testing was at an early stage therefore about 50 individuals were tested nationwide per day with about five testing positive every day. The number of deaths reported was low, at least one every day during our research. At these times, a journalist and a social media user were arrested for “spreading false news” about healthcare system capacities. The official response of the government went from initial jovial narrative and underestimation of the danger to one of serious threat. Similar situation was found with hospital capacities^1^—at first, people were informed that there were just enough capacities encouraging people that Serbia is ready to fight against the virus, while very fast, the information went all the way to panic reports about not having capacities and the need for extra hospitals. This ambiguous information, along with other instances and ever tighter restrictive measures contributed to the prevailing negative atmosphere of that time.

To get more insight in well-being during the pandemic, we decided to control for variables that were proven to have an impact on well-being during the pandemic in previous studies: age, gender, health status, working status, marital status, and having children.

## Materials and Methods

### Participants and Procedure

The study involved 965 adults of COVID-19-free participants from the general population, aged from 17 to 85 (*M*_age_ = 29, 57% females) from Serbia. The greatest proportion of the participants were students (45%), followed by employed (36%), non-employed (8.8%), self-employed (6.4%), homemakers (1.8%), and the least retired people (1.2%). Regarding marital and relationship status, 40.4% are single, 32% are in a relationship, 20.7% are married for the first time, 2.1% are married for the second time, 3.9% are divorced, and 0.8% are widowed. With regard to offspring, 26.3% of our participants have children. The average number of individuals in a participant's household was 3.55; 9.7% of participants indicated that they suffer from a chronic illness. These illnesses were mostly endocrinological and cardiovascular in nature. We have also asked participants about their behavior and attitude toward the pandemic and government measures. These questions are answered on a scale from 1 to 5 with 1 being the negative and 5 the positive pole of the scale. These results suggest that people were quite keen on informing about the virus, were not so much afraid, did have good resources of communication and activities, and have asked for emotional support from their closest, and, in less extent, from mental health care professionals (Table 1).

**Table 1:** Central tendency and dispersion on questions about reactions to the pandemic.

**Items**	**Mean**	**SD**
To what degree did you watch pandemic related content on TV today?	2.44	1.18
To what degree do you follow daily number of deaths and infected from COVID-19?	3.19	1.19
To what degree are your thoughts dominated by the pandemic?	2.67	1.05
To what degree are you frightened for your life?	1.86	1
To what degree have you ventured outside today?	2.25	1.26
To what degree are you satisfied with your communication with members of your household?	4.08	0.98
To what degree are you active in the context of your employment and studying obligations?	3.31	1.34
To what degree are you physically active during the pandemic?	3.07	1.26
To what degree do you feel the need for emotional support from individuals closest to you during the pandemic?	3.16	1.24
To what degree do you feel the need for psychological support in the time of the pandemic?	2.25	1.28

The data were gathered online in the third week after the state of emergency in Serbia was declared. An online questionnaire was administered by first-year and second-year students of psychology at the Faculty of Legal and Business Studies Dr Lazar Vrkatić, in exchange for course credits in courses Methodology of Psychological Research and Educational Psychology.

Ethical approval for conducting this study was obtained from the Ethical Board of the Faculty of Legal and Business Studies Dr. Lazar Vrkatić. The scales were administered to participants using an online format. Participation in the study was completely voluntary and participants could give up at any time.

### Instruments and Variables

1. *The Basic Psychological Needs Satisfaction and Frustration Scale (BPNSFS)* [(50); Serbian adaptation and translation—(54)] was used for assessing autonomy satisfaction (4 items, e.g., I feel that my choices express who I really am; α = 0.74), competence satisfaction (4 items, e.g., I feel capable at what I do α = 0.80), and relatedness satisfaction (4 items, e.g., I feel that people I care about also care about me α = 0.78); and three needs frustration subscales: autonomy frustration (4 items, e.g., I feel forced to do many things I wouldn't choose to do α = 0.76), competence frustration (4 items, e.g., I feel like a failure because of the mistakes I make α = 0.81), and relatedness frustration (4 items, e.g., I feel the relationships I have are just superficial α = 0.71). The scale was validated on a Serbian sample and showed promising psychometric characteristics and a 6-factor solution (54). We asked participants to answer to items regarding the following week using a 5-point Likert scale, ranging from 1 (I completely disagree) to 5 (I completely agree). Mean scores on six scales were treated as mediators in the statistical analysis.
2. *Satisfaction With Life Scale (SWLS)*—[(16); Serbian adaptation and translation—(76)] is a unidimensional scale consisting of five items (e.g., *In most ways my life is close to my ideal*; α = 0.81). SWLS was previously validated in Serbian context proving its good psychometric properties (76). Responses on the items were registered on a 5-point Likert scale from 1 (I completely disagree) to 5 (I completely agree). This scale was used for measuring one of the basic components of subjective well-being—satisfaction with life, that in this study was treated as criterion variable.
3. *The Serbian Inventory of Affect based on the Positive and Negative Affect Schedule-X (SIAB-PANAS)* (77) is a Serbian translation and adaptation of the Positive and Negative Affect Schedule-X (PANAS-X) (78). For this research, we used the short dispositional form designed to measure the traits of Positive Affect (PA) and Negative Affect (NA), with 10 items each. Participants were asked to report how they felt in general, using a 5-point Likert scale ranging from 1 (*never or almost never*) to 5 (*always or almost always*). The scale has demonstrated excellent psychometric properties and good reliability with a Cronbach's alpha of 0.88 and 0.87 in our research, as well as in previous research with a Cronbach's alpha of 0.85 and 0.83 for PA and NA, respectively (79). Mean scores were calculated on PA and NA and served as predictors in performed analysis.
4. *The Depression Anxiety and Stress Scale-21 (DASS-21)* (44) was used to assess negative affective states. The DASS-21 consists of 21 items and includes three subscales of depression, anxiety, and stress. Responses are rated on a 4-point scale, from 0 (*did not apply to me at all*) to 3 (*applied to me greatly, or most of the time*). The scale also provides the general score representing the level of psychological distress and was involved in analysis as criterion variable. The DASS-21 translation into Serbian is widely used and has shown good reliability in our research (α = 0.93) as in previous research (α = 0.92) both in an adult (80) and adolescent sample (81).

Age, gender, health status, working status, marital status, and having children were treated as control variables.

### Data Analytic Strategy

As part of our investigation, we performed descriptive analysis and correlation analysis on the mean scores of the variables included. Mediation analysis was conducted in Hayes' (82) PROCESS macro. We used Model 6 (Graph) that specified a serial multiple mediator model, in which we chained in sequence six mediators—satisfaction and frustration of the basic psychological needs. The PROCESS allows accounting for the total effects of all variables on criterion, direct effects of predictors while controlling for mediators, as well as indirect effects of mediators on criterion variables. In total, four models that included two independent predictors (positive and negative affect), six mediators (satisfaction and frustration of the need for autonomy, competence and relatedness), and two independent criteria (satisfaction with life and general distress) were tested with the aim of examining the sequential influence of six mediators in the hypothesized causal relation, and to verify whether each mediator had an independent effect on the outcomes, while controlling for age, gender, health status, working status, marital status, and having children. Both direct and indirect effects were compared to 95% confidence intervals with Bonferroni correction bootstrapped on 5,000 random data sets.

## Results

Table 2 presents the correlations between the variables used in this study in the status of predictors, mediators, and criterions. All the correlation coefficients are statistically significant and in expected directions. Having satisfied all the conditions for mediation analysis, we proceeded further with testing the direct and indirect relations of the variables. According to the means of the measures, all positively associated aspects of human functioning are greater than negative ones—positive affect is greater than negative affect, satisfaction with life is greater than general distress, and all satisfactions of the needs are greater than their frustrations. It is worth mentioning that relatedness and competence satisfaction is more than twice as pronounced as their frustration, while that difference is less significant in the case of autonomy.

**Table 2:** Correlations between predictors, mediators, and criterion variables (means and standard deviations).

**Scale**		**1**	**2**	**3**	**4**	**5**	**6**	**7**	**8**	**9**	**10**
1.	Positive affect	1									
2.	Negative affect	−0.30^**^	1								
3.	Satisfaction with life	0.38^**^	−0.31^**^	1							
4.	Psychological distress	−0.38^**^	0.71^**^	−0.35^**^	1						
5.	Autonomy satisfaction	0.42^**^	−0.27^**^	0.53^**^	−0.31^**^	1					
6.	Autonomy frustration	−0.24^**^	0.42^**^	−0.30^**^	0.45^**^	−0.42^**^	1				
7.	Competence satisfaction	0.48^**^	−0.28^**^	0.37^**^	−0.37^**^	0.49^**^	−0.25^**^	1			
8.	Competence frustration	−0.42^**^	0.48^**^	−0.38^**^	0.58^**^	−0.36^**^	0.43^**^	−0.59^**^	1		
9.	Relatedness satisfaction	0.26^**^	−0.23^**^	0.41^**^	−0.24^**^	0.46^**^	−0.26^**^	0.31^**^	−0.31^**^	1	
10.	Relatedness frustration	−0.14^**^	0.39^**^	−0.28^**^	0.40^**^	−0.32^**^	0.45^**^	−0.28^**^	0.48^**^	−0.55^**^	1
	M	3.28	2.08	3.56	0.68	3.82	2.62	4.14	1.81	4.44	1.72
	SD	0.74	0.70	0.80	0.55	0.74	0.91	0.61	0.82	0.60	0.70

*M, mean; SD, standardized deviation*;

* *p < 0.05*;

** *p < 0.01*.

**Figure 1:**
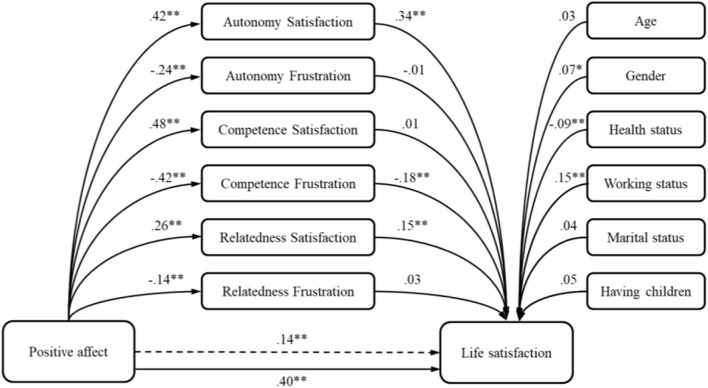
The model of positive affect predicting life satisfaction—the mediation role of basic psychological needs. *p < 0.05; **p < 0.01.

Positive affect can account for 18% of life satisfaction variance. As we can see in Table 3, positive affect demonstrates a significant potential in predicting life satisfaction. However, the subsequent model which included six mediators accounted for 39% of criterion variance, demonstrating greater predictive potential (Δ*R*^2^ = 0.21; *p* < 0.01). As the total indirect effect accounts for 65% of the total effect achieved in the model, the results suggest that the relation of positive affect and life satisfaction is partially mediated by the positive effect of autonomy and relatedness satisfaction, as well as by negative effect of competence frustration (Figure 1).

**Table 3:** Testing the mediation role of basic psychological needs in the relation between positive affect and life satisfaction.

	***B***	***B***	**SE**	***p***	**BC bootstrapping 95%**	
					**LLCI**	**ULCI**
Model 1: *R*/*R*^2^ 0.42/0.18						
Constant	2.01		1.88	0.00	1.53	2.50
Age	−0.01	−0.03	0.01	0.56	−0.01	0.01
Gender	0.12	0.07	0.05	0.02	0.00	0.25
Health status	−0.29	−0.11	0.08	0.00	−0.50	−0.08
Working status	0.06	0.16	0.01	0.00	0.02	0.09
Marital status	0.02	0.03	0.03	0.47	−0.06	0.10
Having children	0.09	0.10	0.04	0.03	−0.02	0.19
Positive affect	0.43	0.40	0.03	0.00	0.35	0.52
Model 2: *R*/*R*^2^ 0.62/0.39						
Constant	0.84		0.30	0.01	0.06	1.61
Age	0.00	0.03	0.00	0.52	0.00	0.01
Gender	0.12	0.07	0.04	0.01	0.01	0.22
Health status	−0.24	−0.09	0.07	0.00	−0.42	−0.05
Working status	0.05	0.15	0.01	0.00	0.02	0.08
Marital status	0.03	0.04	0.03	0.26	−0.04	0.10
Having children	0.04	0.05	0.03	0.20	−0.05	0.13
Positive affect	0.15	0.14	0.03	0.00	0.06	0.24
Autonomy satisfaction	0.37	0.34	0.04	0.00	0.27	0.46
Autonomy frustration	−0.01	−0.01	0.03	0.69	−0.08	0.06
Competence satisfaction	0.01	0.01	0.04	0.80	−0.10	0.12
Competence frustration	−0.18	−0.18	0.04	0.00	−0.27	−0.09
Relatedness satisfaction	0.19	0.15	0.04	0.00	0.08	0.31
Relatedness frustration	0.03	0.03	0.04	0.41	−0.07	0.14
Direct effect	0.15	0.14	0.03	0.00	0.06	0.24
Total indirect effect	0.29	0.26	0.02	0.00	0.21	0.33
Total effect	0.43	0.40	0.03	0.00	0.35	0.52

**Figure 2:**
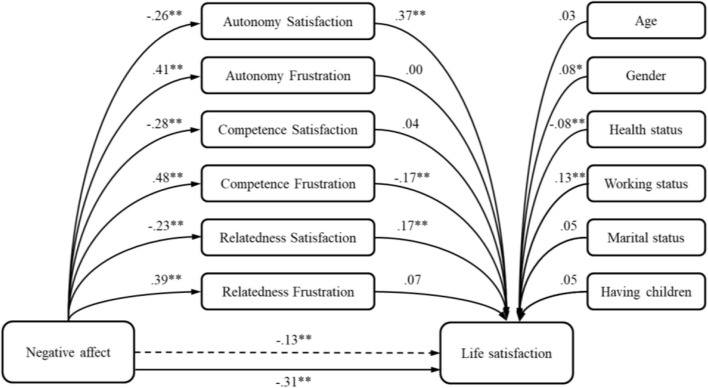
The model of negative affect predicting life satisfaction—the mediation role of basic psychological needs. *p < 0.05; **p < 0.01.

Very similar results were obtained by testing the mediation role of basic psychological needs in relation between negative affect and life satisfaction. Model 1 (Table 4) demonstrated lower but still significant predictive potential of negative affect, which accounts for 14% of life satisfaction variance. By building the sequential model by adding the basic psychological needs significantly raised the percent of explained criterion variance (Δ*R*^2^ = 0.25; *p* < 0.01) but deteriorated the unique predictive potential of negative affect. A 64% of the total effect is achieved through the indirect effect of autonomy satisfaction, relatedness satisfaction, as well as competence frustration (Figure 2).

**Table 4:** Testing the mediation role of basic psychological needs in the relation between negative affect and life satisfaction.

	***B***	***B***	**SE**	***p***	**BC bootstrapping 95%**	
					**LLCI**	**ULCI**
Model 1: *R*/*R*^2^ 0.37/0.14						
Constant	4.09		0.16	0.00	3.68	4.50
Age	−0.01	0.00	0.04	0.38	−0.01	0.00
Gender	0.17	0.10	0.05	0.01	0.04	0.30
Health status	−0.21	−0.09	0.08	0.02	−0.42	0.01
Working status	0.04	0.10	0.01	0.01	0.00	0.07
Marital status	0.05	0.06	0.03	0.15	−0.04	0.13
Having children	0.11	0.12	0.04	0.00	0.00	0.21
Negative affect	−0.18	−0.31	0.02	0.00	−0.46	−0.28
Model 2: *R*/*R*^2^ 0.62/0.39						
Constant	1.06		0.30	0.00	0.26	1.73
Age	0.00	0.03	0.00	0.47	0.00	0.01
Gender	0.13	0.08	0.04	0.00	0.02	0.24
Health status	−0.22	−0.08	0.07	0.00	−0.40	−0.03
Working status	0.04	0.13	0.01	0.00	0.02	0.07
Marital status	0.04	0.05	0.03	0.18	−0.04	0.11
Having children	0.05	0.05	0.03	0.17	−0.04	0.14
Negative affect	−0.14	−0.13	0.02	0.00	−0.23	−0.05
Autonomy satisfaction	0.39	0.37	0.04	0.00	0.30	0.49
Autonomy frustration	0.00	0.00	0.03	0.90	−0.07	0.08
Competence satisfaction	0.05	0.04	0.05	0.48	−0.06	0.16
Competence frustration	−0.16	−0.17	0.04	0.00	−0.26	−0.06
Relatedness satisfaction	0.22	0.17	0.05	0.00	0.10	0.33
Relatedness frustration	0.08	0.07	0.05	0.05	−0.02	0.18
Direct effect	−0.14	−0.13	0.04	0.00	−0.23	−0.05
Total indirect effect	−0.23	−0.20	0.03	0.00	−0.31	−0.15
Total effect	−0.37	−0.33	0.04	0.00	−0.46	−0.28

**Figure 3:**
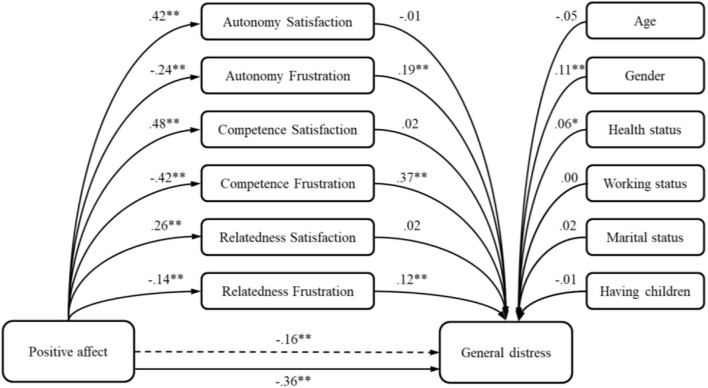
The model of positive affect predicting general distress—the mediation role of basic psychological needs. *p < 0.05; **p < 0.01.

In predicting the general distress, the positive affect also demonstrated a significant negative potential (*R*^2^ = 0.18). By adding the basic psychological needs to the model (Table 5), the percent of explained criterion variance increased (Δ*R*^2^ = 0.25; *p* < 0.01), and the unique predictive power of the positive affect deteriorated but remained significant. Fifty-five percent of the total effect was achieved by an indirect effect of autonomy, competence, and relatedness frustration (Table 5; Figure 3).

**Table 5:** Testing the mediation role of basic psychological needs in the relation between positive affect and general distress.

	***B***	***B***	**SE**	***p***	**BC bootstrapping 95%**	
					***LLCI***	**ULCI**
Model 1: *R*/*R*^2^ 0.43/0.18						
Constant	1.12		0.13	0.00	0.79	1.46
Age	−0.01	−0.06	0.01	0.20	−0.01	0.00
Gender	0.15	0.14	0.03	0.00	0.07	0.24
Health status	0.19	0.10	0.06	0.00	0.05	0.34
Working status	0.01	0.03	0.01	0.35	−0.01	0.03
Marital status	0.02	0.03	0.02	0.42	−0.04	0.07
Having children	−0.02	0.03	0.03	0.47	−0.09	0.05
Positive affect	−0.27	−0.36	0.02	0.00	−0.33	−0.21
Model 2: *R*/*R*^2^ 0.66/0.43						
Constant	−0.21		0.20	0.30	−0.72	0.31
Age	0.01	−0.05	0.01	0.20	−0.01	0.00
Gender	0.12	0.11	0.03	0.00	0.05	0.19
Health status	0.11	0.06	0.05	0.02	−0.01	0.23
Working status	0.00	0.00	0.01	0.88	0.05	0.02
Marital status	0.01	0.02	0.02	0.64	−0.04	0.06
Having children	−0.01	−0.01	0.02	0.81	−0.07	0.05
Positive affect	−0.12	−0.16	0.02	0.00	−0.18	−0.06
Autonomy satisfaction	−0.01	−0.01	0.02	0.69	−0.07	0.05
Autonomy frustration	0.12	0.19	0.02	0.00	0.07	0.16
Competence satisfaction	0.02	0.02	0.03	0.56	−0.06	0.09
Competence frustration	0.25	0.37	0.02	0.00	0.18	0.31
Relatedness satisfaction	0.02	0.02	0.03	0.57	−0.06	0.09
Relatedness frustration	0.09	0.12	0.03	0.00	0.02	0.16
Direct effect	−0.12	−0.16	0.02	0.00	−0.18	−0.06
Total indirect effect	−0.15	−0.19	0.03	0.00	−0.26	−0.10
Total effect	−0.27	−0.35	0.02	0.00	−0.33	−0.21

**Figure 4:**
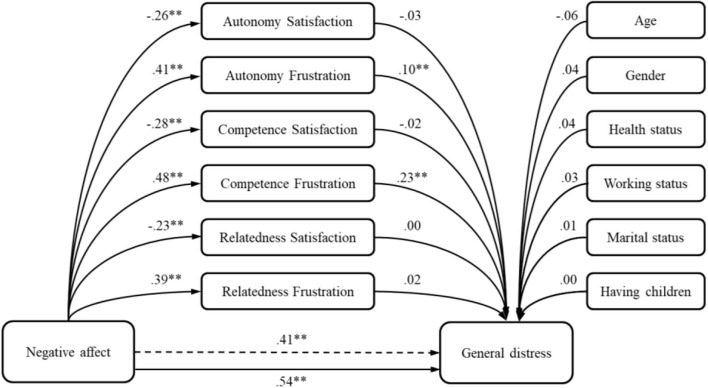
The model of negative affect predicting general distress—the mediation role of basic psychological needs. **p < 0.01.

In the fourth model tested, the negative affect accounted for 52% of general distress variance. By adding the basic psychological needs to the model, the total explained variance of the criterion increased (Δ*R*^2^ = 0.08; *p* < 0.01). As we can see from Table 6 and Figure 4, the relation between the negative affect and general distress is partially mediated by autonomy and competence frustration.

**Table 6:** Testing the mediation role of basic psychological needs in the relation between negative affect and general distress.

	***B***	***B***	**SE**	***p***	**BC bootstrapping 95%**	
					**LLCI**	**ULCI**
Model 1: *R*/*R*^2^ 0.72/0.52						
Constant	−0.62		0.08	0.00	−0.83	−0.41
Age	0.00	−0.06	0.00	0.11	−0.01	0.00
Gender	0.04	0.04	0.03	0.09	−0.02	0.11
Health status	0.09	0.05	0.04	0.05	−0.02	0.20
Working status	0.02	0.08	0.01	0.00	0.00	0.04
Marital status	0.01	0.01	0.02	0.84	−0.04	0.05
Having children	−0.01	−0.02	0.02	0.50	−0.07	0.04
Negative affect	0.54	0.69	0.02	0.00	0.50	0.59
Model 2: *R*/*R*^2^ 0.77/0.60						
Constant	−0.56		0.17	0.00	−1.00	−0.13
Age	0.00	−0.06	0.00	0.07	−0.01	0.00
Gender	0.05	0.04	0.00	0.04	−0.01	0.11
Health status	0.07	0.04	0.02	0.07	−0.03	0.18
Working status	0.01	0.03	0.04	0.19	−0.01	0.02
Marital status	0.00	0.01	0.01	0.79	−0.04	0.04
Having children	0.00	0.00	0.02	0.92	−0.05	0.05
Negative affect	0.41	0.52	0.02	0.00	0.36	0.46
Autonomy satisfaction	−0.03	−0.03	0.02	0.21	−0.08	0.03
Autonomy frustration	0.06	0.10	0.02	0.00	0.02	0.10
Competence satisfaction	−0.02	−0.02	0.02	0.38	−0.08	0.04
Competence frustration	0.15	0.23	0.02	0.00	0.10	0.21
Relatedness satisfaction	0.01	0.00	0.03	0.94	−0.07	0.06
Relatedness frustration	0.02	0.02	0.02	0.45	−0.04	0.08
Direct effect	0.41	0.52	0.02	0.00	0.36	0.46
Total indirect effect	0.14	0.17	0.02	0.00	0.10	0.18
Total effect	0.55	0.69	0.05	0.00	0.50	0.59

## Discussion

Has well-being changed during the COVID-19 pandemic? How did people feel and how did that relate to their sense of satisfaction with life? Did the current situation, that involved state of emergency and a potential threat on life, affect the general distress? Moreover, how did the pandemic affect the basic psychological needs that are crucial for attaining the well-being? A theoretical framework that has a clear guideline on these questions is the Self-Determination Theory (20, 46), which claims that basic psychological need satisfaction is critical in achieving greater well-being, while their frustration is responsible for ill-being.

The general aim of this research was to examine well-being during the outbreak of COVID-19. We examined the relationship between positive and negative affects and satisfaction with life and distress and surveyed the role of basic psychological needs as mediators and mechanisms of achieving well-being. Given the fact that well-being is highly dependent on social circumstances (55), it is important to accentuate that study was performed in the third week after state of emergency was declared Serbia, which has brought quite a negative atmosphere in population due to many ambiguous information gained by the government.

In achieving our aim, we tested four models in which positive and negative affects were predictors, satisfaction with life and general distress were criterion variables, while satisfaction and frustration of the basic psychological needs were involved as mediators, controlling for age, gender, health status, working status, marital status, and having children. Results showed that both positive and negative affects demonstrate a significant potential in predicting the life satisfaction. Thus, when evaluating their satisfaction with life during the pandemic, people tend to rely on both their positive and negative experiences and emotions. In comparison with positive affect, negative affect had a weaker relationship with life satisfaction. Our results are in line with some previous research which indicated that both positive and negative affects are important in evaluating the satisfaction with life (30). However, in our research, the strength of the effects of the positive and negative affects were similar [like in (32)], while Kuppens et al. (30) revealed that negative affect had an effect that was twice weaker than positive affect's effect on satisfaction with life. This difference could be explained by the context of the pandemic, that people in the times of an actual threat take into account both negative and positive feelings more equally since they are more prominent than in “normal” times (6). Negative feelings might have been more pronounced during the actual week of the research, when many uncertainties were present and people did not have enough information about the actual threat nor about the future life circumstances (questions like are there enough hospital capacities, enough tests, enough medical personnel, etc.). Also, we could explain the effect of negative affect on satisfaction with life by cultural differences (30). We could argue that in a country as Serbia, where the satisfaction with life is lower than in other more economically developed countries (76), people focus less on their self-expression and on satisfaction of their psychological needs (34), and more on their basic and economic needs, thus negative affect is expected to be taken more into considerations when evaluating personal satisfaction with life (30). This was likely even more pronounced in the pandemic situation when people had hard times believing in the government when giving the ambiguous COVID-19-related information.

Furthermore, relations between the affects and satisfaction with life were even stronger when the needs for autonomy and relatedness were satisfied and when competence was not thwarted. This result was somewhat expected, since previous studies (50, 60–63) as well as SDT postulates (20) indicated that satisfaction of the basic psychological needs is an important salient of the satisfaction with life, as one aspect of subjective well-being.

Among the unique contributions of basic psychological needs, autonomy satisfaction had the greatest indirect effect on satisfaction with life, in the models with both positive and negative affects. This was followed by the mediating effect of relatedness satisfaction, which had lower indirect effect, and competence frustration had the smallest effect. When government brought measures that are unpopular and freedom constraining, especially if they were communicated in a controlling manner (83), as they were likely in Serbia, people may have even had a greater need to express their own opinions and make their own choices. Also, satisfaction with life was dependent on relatedness satisfaction, probably because “staying at home” gave people the opportunity to stay with their families. However, the effect of the relatedness satisfaction was lower than the effects of the other two needs satisfaction.

In the only found study that tapped into the relations between the basic psychological needs and well-being in the pandemic so far, by Cantarero et al. (69), the need for competence was the most important predictor of the well-being, relatedness satisfaction was also significant, while autonomy was not. This difference in results might stem from different measures enacted against the pandemic in the two countries or from the severity of the coronavirus outbreak in the two countries as well as from some culture differences (84). However, further research would be needed to look these differences more thoroughly.

In general, from obtained results, we can say that satisfaction with life during the pandemic can be enhanced when satisfying foremost the need for autonomy and the relatedness need as well as lowering the competence frustration, in both those who were more disposed to feel more positively or more negatively. These basic psychological needs play as mechanisms through which satisfaction with life can be obtained during the pandemic.

In predicting general distress, obtained results revealed that both positive and negative affects have significant direct relations. Positive affect demonstrated a significant negative potential in explaining general distress, and it has diminished by adding the mediators, but it still remained significant. The significance of the frustration of the three needs in the relations between positive affect and general distress was expected, since the frustration of the needs is convergent to the nature of ill-being, as revealed in previous studies (48, 50, 64, 65). This means that positive emotions are a protective factor for feeling anxious, depressive, and stressed, but when the basic needs are frustrated, distress increases. During the first weeks of the pandemic in Serbia, which were filled with uncertainty, this result meant that those people who entered the pandemic with more positive feelings and optimism, had lower levels of depression, anxiousness, and stress, but when basic psychological needs were thwarted due to the pandemic context, even those who felt more positively were more prone to depression, anxiety, and stress.

Explaining 50% of the explained variance of general distress, negative affect is a much stronger predictor of distress than positive affect. Similar result was found in previous studies (27, 39, 41–43). Furthermore, this relation was even higher when mediators were involved, but their unique indirect effects were not so high, due to the strong influence of the predictor itself. Competence frustration had the greatest indirect effect, while autonomy frustration had an effect of only 0.10. This result indicates that when the sense of competence is thwarted within the environment, distress is more pronounced. Those individuals who entered the pandemic feeling more negatively suffered even more distress during the crisis, and especially in those individuals who felt incompetent and controlled. Theoretically, this result was expected since it is a probable consequence of the nature of the phenomena examined, which are highly overlapping and cover a wide range of mutual characteristics.

Since the frustration of the need for competence comprises the sense of personal failure or inadequacy (48), we could argue that this feeling was the most important mediator in tested model due to the change of life during the pandemic. As proposed in the SDT, thwarting environment could diminish the satisfaction of the needs and increase its frustration, we could argue that in the times of the pandemic, which brought a sense of uncertainty and fear of the insufficiently understood threat, as well as increased stress and depression (6), it has also influenced the feeling of inadequacy or personal failure (48). Furthermore, the current situation was a worldwide and also a threat on the national level, leaving to individuals just to “stay at home.” This strategy might have triggered learned helplessness, where people developed a belief that they cannot influence the current situation and that could have led to a withdrawing and passive behavior (85–87).

### Practical Implications

Our results indicated that in order for people to remain stable in terms of well-being during the pandemic, it is important that the basic psychological needs remain satisfied. These findings could be of importance for decision-makers who are responsible for national health issues, including mental health as well. Thus, several practical implications on how to deal with the basic psychological needs in the times of crisis are discussed. In order to maintain optimal autonomy satisfaction, governments could, when in need of introducing unpopular measures in order to save lives, announce their measures in an autonomously supporting ways, so that people find them useful, internalize their value and adhere to proscribed measures. Giving autonomy supporting messages about the measures for social distancing and for staying at home, as a preventive measure, by explaining the value of the behavior, could help people understand and therefore internalize the value of the change in behavior (83). On the contrary, controlling messages that use shame, induce guilt, and a must, can have a negative effect in terms of even backfiring and increasing the undesired behavior change (88). Furthermore, in order to help maintain optimal relatedness satisfaction, other options for live contact could be promoted, such as virtual meetings, virtual cafés, etc. Lastly, giving people opportunity to volunteer and help others in various ways in order to stay active could help people feel satisfied with their competence during the pandemic.

### Advantages and Limitation of the Study

Research on the pandemic is ongoing and will probably stay relevant for some time in the future. Up to now, after a few months from the beginning of the pandemic, research have revealed an increase of anxiety, depression, stress, and lower well-being (4–6, 8) in women, younger people, parents, and those with chronical disease (7, 8, 11, 12). However, up to date, there is no knowledge about how do these consequences of the pandemic develop. Our study reveals an important question on mechanisms of how does well-being change in people disposed to feel more positive and more negative. To our knowledge, this study is unique in investigating the mediation role of both satisfaction and frustration of basic psychological needs within the relations of dispositional affect and indicators of well-being, using the serial multiple mediation modal. Furthermore, our research adds to the COVID-19-related research on how people felt during the pandemic in Serbia, while there are many research on the pandemic issues in other, usually more developed parts of the world. Thus, our research adds to the literature on how did a country of lower economic status deal with the pandemic and how did its people feel.

The COVID-19 pandemic as a general negative life event situation does not only provided situational relevance but also reduced the external validity of our findings. Replicating our research out of the pandemic context is highly recommended in order to differentiate the situational effect from the general mediation role of basic psychological needs. Since culture can have a moderating effect on the examined relations in our study, a cross-cultural study would be needed. Furthermore, since the pandemic lasted at least 3 months and is still in fact active, and that certain countries have been changing their measures regarding the pandemic following the pandemic curve of development, a longitudinal design would be appropriate to check for the trajectories in well-being. Moreover, due to the complexity of our study design, we did not take into account the socio-demographic characteristics of the sample, we held them under control, and since there are evidence about the greater distress in female gender (8–11) and people with chronical disease (7, 11), in parents balancing between personal life and work (12), future research could also include these variables.

## Conclusion

Results obtained suggest that satisfaction and frustration of basic psychological needs have a significant mediating effect in explaining the relations between affects and well-being during the pandemic in Serbia. Satisfaction with life during the pandemic, as one component of well-being, can be enhanced by satisfying foremost the need for autonomy and relatedness as well as lowering competence frustration, in both individuals who were disposed to feel more positively or more negatively. Furthermore, people who entered the pandemic with more positive feelings and optimism had felt lower levels of general distress, but when basic psychological needs were thwarted due to the pandemic context, even those who felt more positively were more prone to depression, anxiety, and stress. Those individuals who entered the pandemic feeling more negatively had even more distress during the crisis, and especially in those individuals who felt incompetent and controlled, due to competence and autonomy frustration. These results, coherent with the Self-Determination Theory postulates, add to the understanding of human functioning in the times of extraordinary circumstances during a pandemic, by suggesting that satisfaction and frustration of basic psychological needs might have a key role in obtaining optimal well-being.

## Data Availability Statement

The raw data supporting the conclusions of this article will be made available by the authors, without undue reservation.

## Ethics Statement

The studies involving human participants were reviewed and approved by Ethical Board of the Faculty of Legal and Business Studies Dr. Lazar Vrkatić, 204/20. The patients/participants provided their written informed consent to participate in this study.

## Author Contributions

DŠ, DŽ, and NR contributed to conception and design of the study. NR organized the database. DŠ and DŽ performed the statistical analysis and wrote the results section. DŠ wrote the first draft of the manuscript. All authors contributed to manuscript revision, read, and approved the submitted version.

## Conflict of Interest

The authors declare that the research was conducted in the absence of any commercial or financial relationships that could be construed as a potential conflict of interest.

## Footnotes

^1^Health care capacities of the Republic of Serbia are defined by Government bylaws on this subject from 2020. It states that the healthcare system of Serbia encompasses 38,611 hospital beds, that is 5.5 beds per every 1,000 inhabitants. The JuŽna Bačka country, from where most of the research participants are, has 14 primary health care centers, 6 secondary health care centers, and a tertiary health care center, its university clinic. In total, there are 3,048 hospital beds registered, out of whom 1,425 are located in the university hospital clinical center.
